# Supplementary material for: Genome Identification and Expression Profiling of the PIN-Formed Gene Family in Phoebe bournei under Abiotic Stresses
Source: Int J Mol Sci. 2024 Jan 25;25(3):1452. doi: 10.3390/ijms25031452 (PMC10855349; doi:10.3390/ijms25031452)
Supplement: Supplementary file 1 [file ijms-25-01452-s001.zip › Table-information.pdf]

Table S1. The expression of the 13 *PbPIN* genes in various tissues, including root bark, root xylem, stem bark, stem xylem, and leaf.
Table S2. Information of primers.
Table S3. The predicted secondary structure of PbPINs.
